# Surgical treatment of locked posterior shoulder dislocations with modified McLaughlin procedure: Case series and review of treatment possibilities of reverse Hill-Sachs defect

**DOI:** 10.1016/j.cjtee.2024.09.010

**Published:** 2026-04-09

**Authors:** Ilja Chandoga, Dávid Debnár, Katarína Chandogová, Miroslav Kilian

**Affiliations:** a 2nd Orthopaedics and Traumatology Department, Faculty of Medicine, Comenius University and University Hospital, Bratislava, 85107, Slovakia; b Faculty of Health and Social Work, St. Ladislav, University of Health and Social Work, St. Elizabeth, Nové Zámky, 94034, Slovakia; c Department of Trauma Surgery, Faculty of Medicine, Slovak Medical University and University Hospital, Bratislava, 83101, Slovakia

**Keywords:** Posterior shoulder dislocation, Locked dislocation, Reverse Hill-Sachs defect, McLaughlin procedure

## Abstract

The aim is to draw attention to the presence of posterior traumatic glenohumeral dislocations of the shoulder joint, which may sometimes present as missed injuries. The retrospective study reports a cohort of 9 patients operated on between 2010 and 2020 due to locked posterior traumatic dislocation of the shoulder joint with the reverse Hill-Sachs defect. The modified McLaughlin procedure was performed in all cases. No failure of the reconstruction was observed. Removal of fixation screws was performed in 1 patient due to migration. The average Constant-Murley score during the mean follow-up period of 5.7 years resulted in 76 points. Comparing the relationship between angles characterising the location of the reverse Hill-Sachs defect, the defects enlarge toward the anterior margin. Non-anatomical reconstruction with lesser tubercle transfer results in satisfactory outcomes in reverse Hill-Sachs defects involving up to 35% of the articular surface of the humeral head.

## 1 Introduction

The posterior type of the traumatic glenohumeral dislocation accounts for only 2% − 4% of cases.^1^^,^^2^ It must be distinguished from the posterior dislocation in non-traumatic glenohumeral instability, which is usually present in hypermobility patients as a part of multidirectional instability.^3^ Cases of surgical neck fractures with dorsal dislocation of the humeral head are reported similarly.^4^

In addition to the variable soft tissue pathology of the glenoid labrum or glenohumeral ligaments, the typical traumatic posterior glenohumeral dislocation is characterized by an impaction osteochondral fracture of the anterior surface of the humeral head. This so-called reverse Hill-Sachs defect (RHSD) is at risk of an engagement within the posterior glenoid rim, resulting in locked dislocation. It occurs in up to 50% of cases.^2^^,^^5^ Saupe et al.^6^ reported this injury in 31 out of 36 patients. The size of the defect is the key factor for locking of the humeral head behind the glenoid and usually progresses over time.

Most of these injuries occur during falls. In another 30% of cases, a muscle contraction from epileptic seizures or electric shock, often with bilateral manifestation, is reported.^7^ Approximately 50% of these injuries are not diagnosed at the onset due to variable clinical presentation. The correct diagnosis is often delayed for weeks or months.^8^^,^^9^

The initial X-ray examination may be challenging due to the absence of the sub-glenoid position of the humeral head, as present in anterior dislocations. The distance from the anterior glenoid rim to head convexity over 6 mm suggests the lateralization of the humeral head due to its posterior position and is known as “rim sign".^1^ Over time, even a relatively small RHSD may increase in size, causing the humeral head to become locked. In these cases, the X-ray often shows double convex contours of the humeral head, representing anterior and posterior margins of the RHSD. CT or MRI is the diagnostic methods of choice to determine the size and location of the defect and the simultaneous injury of the glenoid fossa as well. MRI reveals posterior labral and capsular injuries in 60% and, rotator cuff tears in 13% − 20% of cases.^6^^,^^9^

The treatment of traumatic dorsal dislocations depends on the time of the diagnosis and the size of the RHSD. The closed reduction is usually possible in acute cases, with a small defect. In cases of neglected injuries with progression of the defect, the surgery is indicated.

## 2 Methods

The retrospective single-centre study of 9 patients with the locked traumatic posterior glenohumeral dislocation operated on between 2010 and 2020 is reported. The shoulder injury occurred in all patients due to a fall. All patients underwent CT or MRI examination before surgery to confirm the dislocation and to evaluate the defect size. There were 2 women and 7 men in the study group. The mean age was 47.7 years (range 23 − 76 years). The mean size of the defect was 29.7% of the articular surface of the humeral head (range 21% − 35%).

**Fig. 1:**
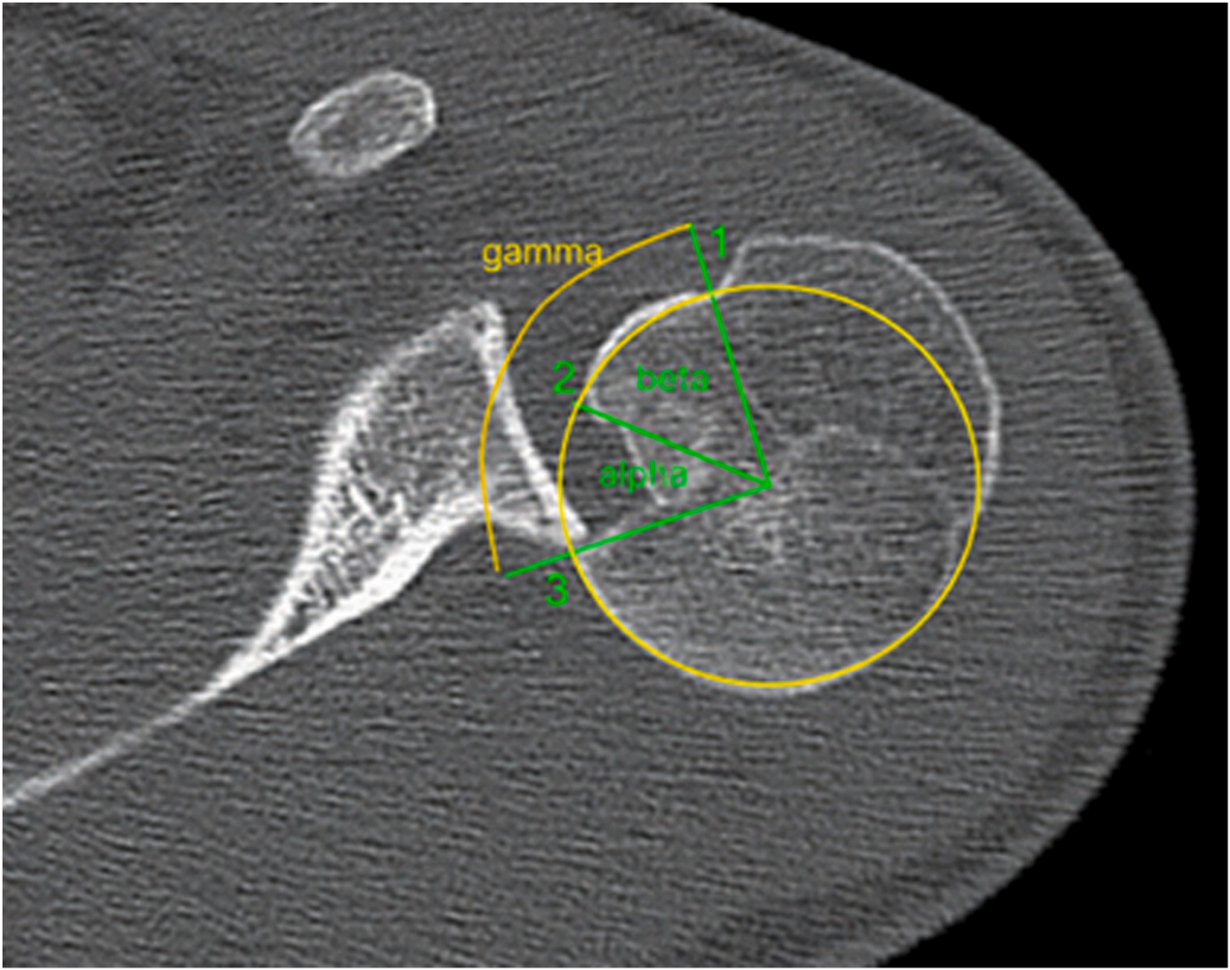
CT scan of the left shoulder joint with locked posterior dislocation. The alpha angle is measured between the anterior (2) and posterior (3) margins of the RHSD and characterizes the defect size. The beta angle is measured between the intertubercular sulcus (1) and the anterior margin (2) of the defect. The gamma angle is measured between the intertubercular sulcus (1) and the posterior margin (3) of the RHSD and represents the sum of alpha and beta angles. RHSD: reverse Hill-Sachs defect.

Patients were treated as locked dislocations with a mean delay after injury of 6.1 weeks (ranging 2 − 15 weeks). In one patient, with a short delay of 2 weeks from the injury and a minor defect size of 21% of the articular humeral head surface, the closed reduction in general anaesthesia was successful, although the glenohumeral instability persisted. The non-operative treatment was abandoned, and open reduction with defect reconstruction was performed in the same setting. In the remaining patients, a closed reduction was not possible, and at the same time, a defect size greater than 20% precluded the possibility of a conservative approach. All patients were indicated for non-anatomical reconstruction by transposition of the lesser tubercle and its fixation into the defect with metallic screws (modified McLaughlin procedure). Measurements of 3 angles characterizing the defect and its relationship to the intertubercular sulcus, as described by Moroder, were performed.^10^ The mean alpha angle defining the defect size and measured between the anterior and posterior margin of the defect was 53° (range 36°− 64°). The beta and gamma angles were measured from the intertubercular sulcus in both cases, toward the anterior margin of the defect in the beta angle, and toward the posterior margin of the defect in the gamma angle (Fig. 1). The mean beta angle was 39.4° (range 30° − 56°) and the mean gamma angle was 92.4° (range 88° − 98°). These angles represent the size and position of the RHSD. The gamma angle over the value of 90° is considered to be a significant risk factor for locking of the dislocation.^11^ It might be expected that the gamma angle would enlarge with the size of RHSD represented with the alpha angle. Patients were followed from 2 to 12 years (mean 5.7 years). The range of motion of the shoulder joint was assessed preoperatively and at the end of the follow-up period, represented with shoulder elevation and external rotation in degrees. Internal rotation of the shoulder joint was assessed by bringing the hand behind the back to the highest vertebral level. Preoperative and follow-up functional outcome of the shoulder joint was evaluated using the Constant-Murley (CM) score.

### 2.1 Surgical procedure

**Fig. 2:**
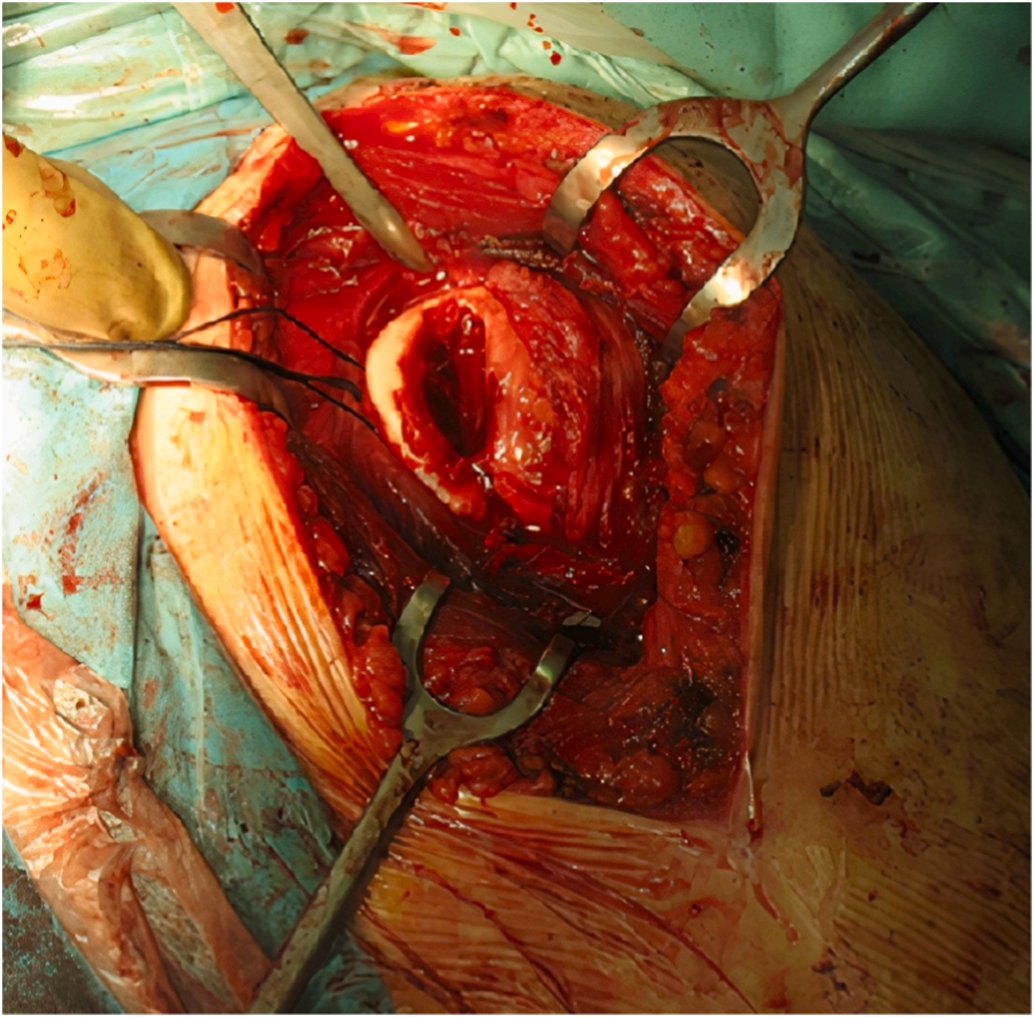
Reverse Hill-Sachs defect of the left humeral head operated on through the deltopectoral approach in a 33-year-old male patient.

**Fig. 3:**
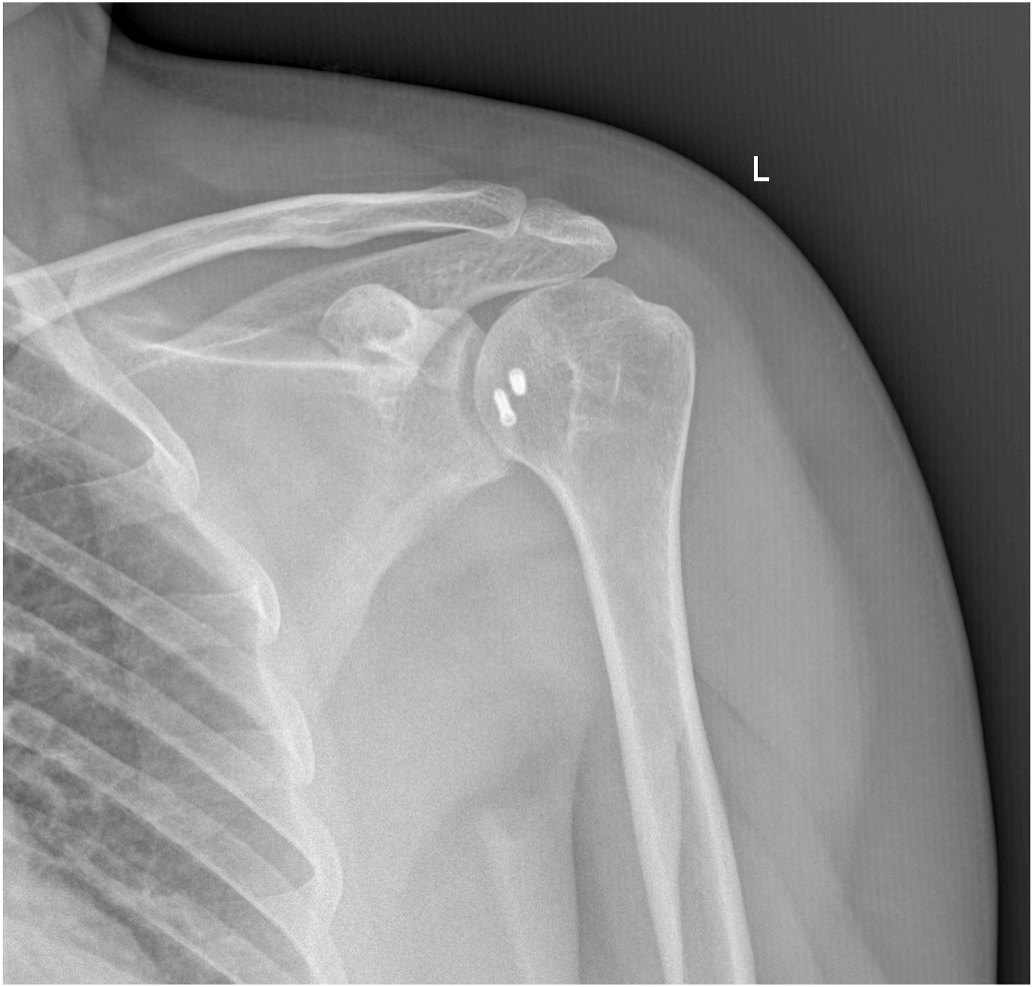
Anteroposterior X-ray of the left shoulder in a 28-year-old woman after the Modified McLaughlin procedure, fixation with 2 cannulated screws.

**Fig. 4:**
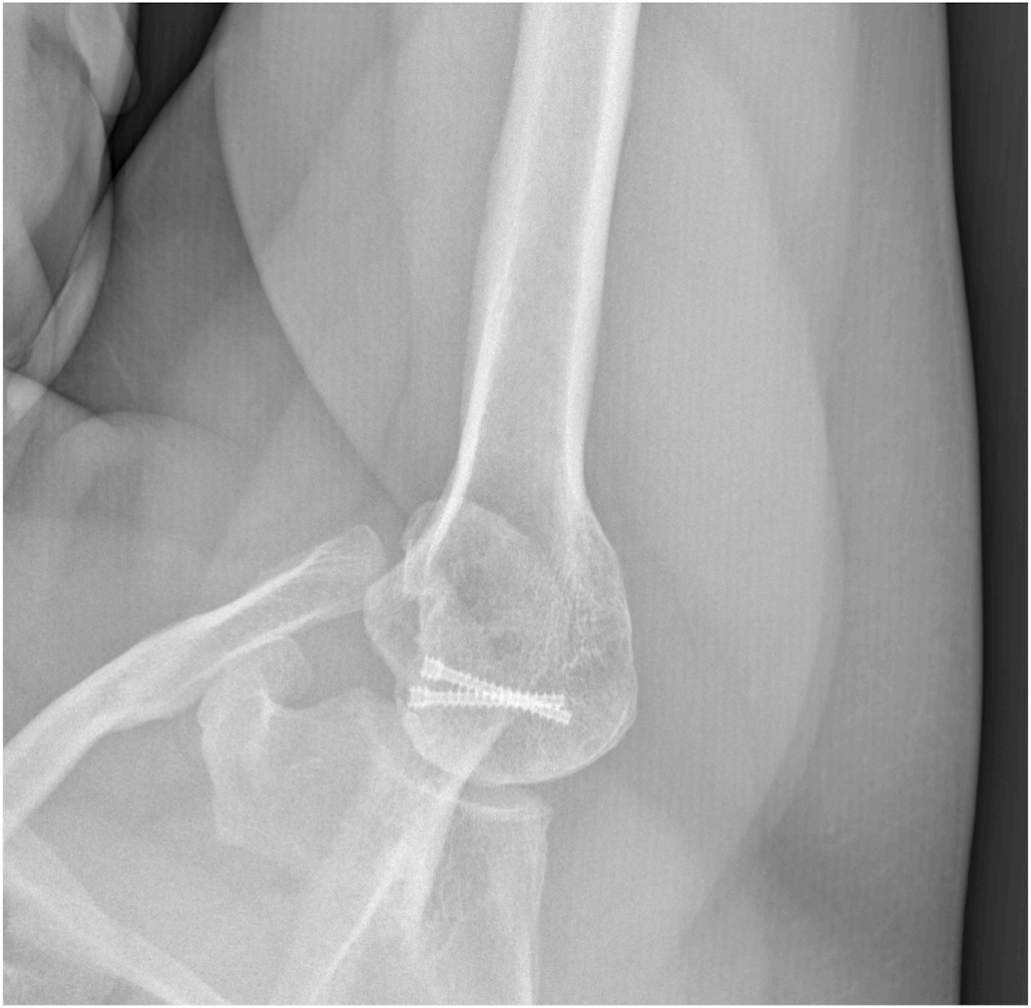
Axial X-ray of the left shoulder in a 28-year-old woman after the modified McLaughlin procedure, fixation with 2 cannulated screws.

The surgery was performed using a deltopectoral approach in a beach chair position. The long head of the biceps tendon was identified, and the osteotomy of the lesser tuberosity was performed with a saw. The complex of the lesser tuberosity and the subscapularis muscle was then reflected medially, and anterior capsulotomy was completed. An open reduction of the humeral head was performed after approaching the glenohumeral joint. The RHSD was then decorticated with a surgical spoon and burr (Fig. 2). The complex of the lesser tuberosity and the subscapularis muscle tendon was then fixed into the defect using cannulated metallic screws (Fig. 3, Fig. 4). Postoperatively, the patient's upper limb was immobilized in neutral rotation for 6 weeks. Physiotherapy followed by isometric exercises, a gradual full range of motion, and passive and active movements.

### 2.2 Statistical analysis

The statistical analysis was performed using the Scientific Python Development Environment (Spyder IDE 5.3.3). Descriptive statistics are reported as the mean with the range. The Shapiro-Wilk test was used to test the normality of the data (the angles, range of motion variables, and CM score). Pearson's correlation coefficient was performed to investigate the relationship between angles characterizing edges of the RHSD. The Wilcoxon signed-rank test was used to compare preoperative and postoperative values. The values of *p* < 0.05 were considered significant.

## 3 Results

**Fig. 5:**
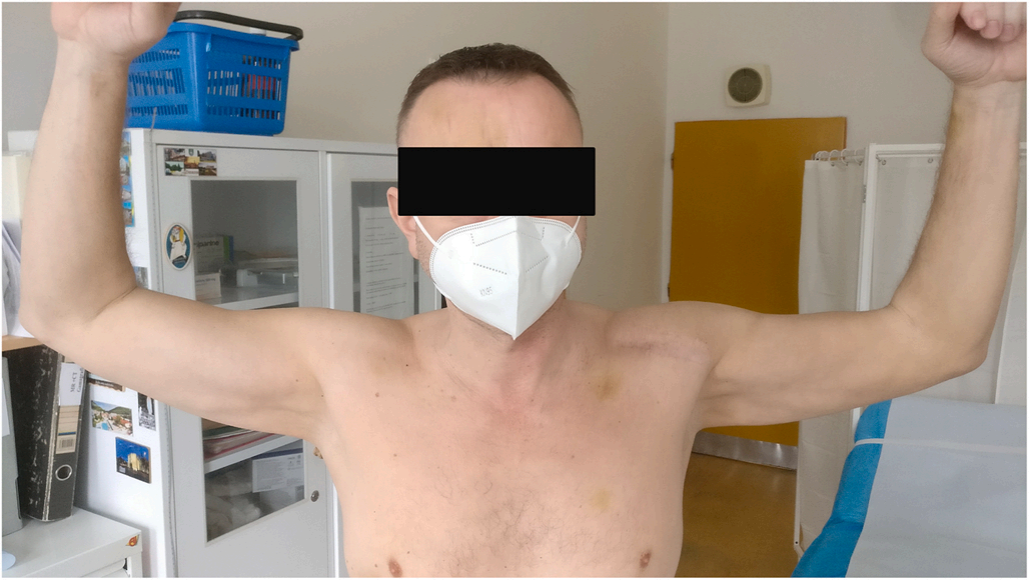
Postoperative external rotation in a 46-year-old male patient 2 years after the surgery.

**Fig. 6:**
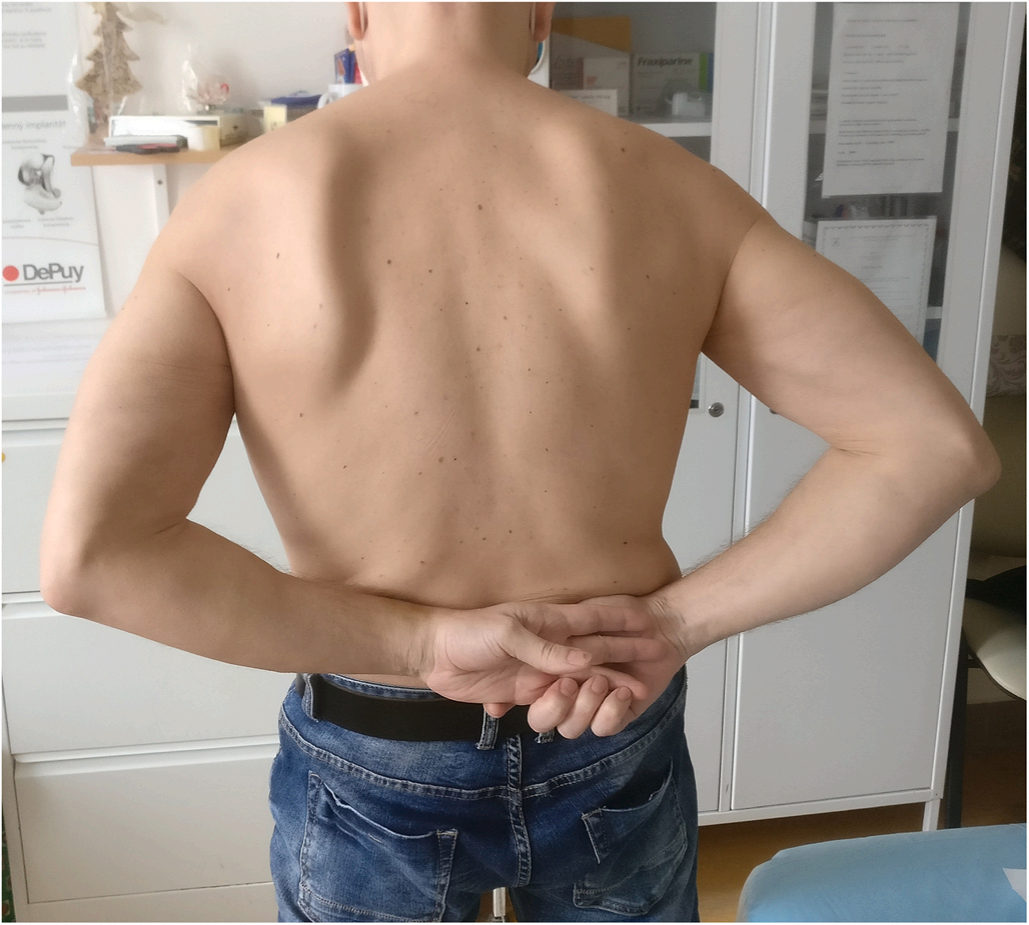
Postoperative internal rotation in a 46-year-old male patient 2 years after the surgery.

**Fig. 7:**
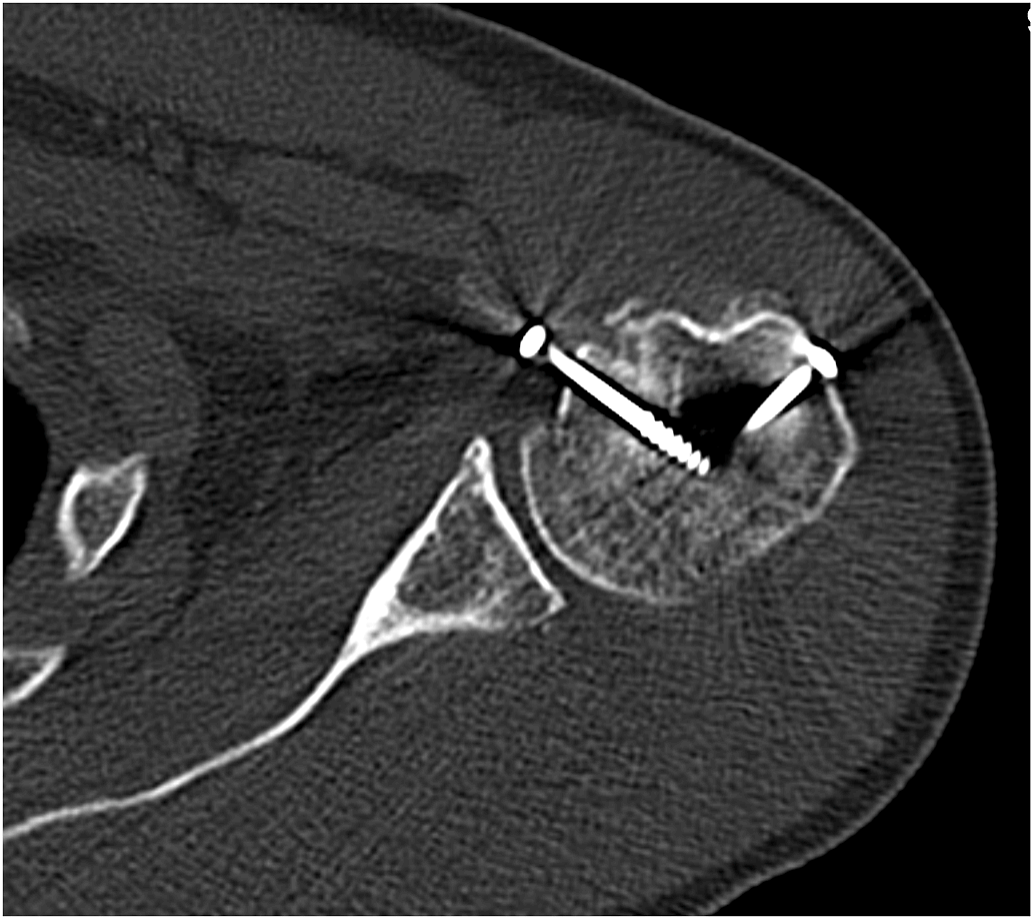
Transverse CT showing screw migration 2 months after the surgery in a 46-year-old man, with the need for surgical removal.

A significant inverse correlation was found between the size of the alpha angle (defect size) and the beta angle, characterising the anterior margin of RHSD (*p* < 0.001). No correlation was found between the size of RHSD (alpha angle) and the gamma angle, which characterizes the posterior margin of the defect (*p* = 0.440) (Table 1). The average CM score in 9 locked cases was 76 points (ranging 67 − 84 points) after a minimum of 2 years of follow-up. Significant improvement of CM score from preoperative middled 37 points was observed, comprehending both elevation and external rotation (*p* = 0.007 and *p* = 0.008, respectively) (Table 2) (Fig. 5). Internal rotation was successfully restored in all patients, and they were able to position the hand of the operated limb, at minimum, to the sacral area (Fig. 6). In one patient, screw migration occurred 2 months after surgery (Fig. 7). Surgical removal of 2 out of 3 fixation screws was indicated. No subjective signs of shoulder joint instability were reported, and there were no septic or surgical incision-related complications. Radiographic evaluation showed no signs of malposition of the humeral head, such as subluxation or luxation, during the follow-up period.

**Table 1:** List of 9 patients showing their personal data and RHSD characteristics before surgery (measurements of angles characterizing the defect size and its location, defect size as a percentage of the articular surface of the humeral head). Relationship between RHSD size (alpha angle) and beta and gamma angles characterizing the edges of the RHSD.^a^

Case	Gender	Age (year)	Delay from injury (week)	Alpha angle (°) (defect size)	Beta angle (°)	*p* value	Gamma angle (°)	*p* value	Defect size (%)
1	male	23	2	36	52		88		21
2	male	76	5	58	31		89		34
3	male	33	2	56	37		93		31
4	female	55	6	61	31		92		34
5	female	28	8	62	36		98		34
6	male	69	13	50	42		92		28
7	male	54	15	42	56		98		23
8	male	46	2	64	30		94		35
9	male	45	2	48	40		88		27
Median (range)		46 (23 − 76)	5 (2 − 15)	56 (36 − 64)	37 (30 − 56)	< 0.001	92 (88 − 98)	0.4400	31 (21 − 35)

RHSD: reverse Hill-Sachs defect.

a Data are presented as median (range) in the final row, and Pearson's correlation coefficient.

**Table 2:** Comparison of pre-OP and FU values of elevation, external rotation, internal rotation, and CM score. Internal rotation was assessed by the highest vertebral level reached with the hand behind the back and converted into an ordinal score (2 – 10 points).^a^

Case	Gender	Age (year)	FU period (year)	Elevation (°)		*p* value	External rotation (°)		*p* value	Internal rotation (°)		*P* value	CM score (points)		*p* value
				Pre-OP	FU		Pre-OP	FU		Pre-OP	FU		Pre-OP	FU	
1	male	23	8	90	140		10	60		LS	T12		35	82	
2	male	76	6	80	110		10	50		B	L5		36	76	
3	male	33	12	90	130		0	60		LS	T12		40	77	
4	female	55	8	100	130		5	50		LS	L3		37	69	
5	female	28	7	110	140		5	70		B	L3		36	74	
6	male	69	2	80	100		0	45		LS	L5		37	67	
7	male	54	4	90	140		10	60		B	L3		35	70	
8	male	46	2	110	155		10	80		LS	L3		40	82	
9	male	45	2	120	160		10	70		LS	L3		37	84	
Median (Q_1_, Q_3_)		46 (33, 55)	6 (2, 8)	90 (90, 110)	140 (130, 140)	0.007	10 (5, 10)	60 (50, 70)	0.008			0.004	37 (36, 37)	76 (70, 82)	0.008

pre-OP: preoperative; FU: follow-up; CM: Constant-Murley; LS: Lumbar Spine; B: Buttock.

a Wilcoxon signed-rank test.

## 4 Discussion

Posterior dislocation of the shoulder joint is a rare injury with a significant proportion of neglected cases, requiring complicated treatment and resulting in uncertain clinical outcomes. The conservative treatment is indicated in case of small RHSD from 10% to 15% of the humeral head cartilage, after a successful closed reduction and full stability when examining the range of motion.^12^^,^^13^ The recommended upper arm immobilisation in our department involved a rigid adjustable orthosis in a slight extra-rotation of around 15°, in order to relieve the RHSD area and at the same time to reduce the tension in the posterior capsule.

Larger defects are most often accompanied by locking of the humeral head, and open reduction and surgical reconstruction of the RHSD is mandatory. Even in acute cases, closed reduction is usually unsuccessful. All patients in our sample had falls as the cause of injury, and there were no patients with non-contact injuries from electric shocks or epileptic seizures. In a multicentre study of 102 patients, Moroder divided traumatic posterior dislocations into non-locked dislocations, acute locked, and chronic locked dislocations. Specifically, in the group of 34 non-locked dislocations, the majority of patients had recurrent posterior instability and minor trauma. On the other hand, locked dislocations were caused by falls and convulsive seizures as well.^14^ In general, it needs to be noted that every RHSD must have shown some type of engagement with the posterior glenoid rim during its formation. The most important of angles characterising the defect, as described by Moroder, is the gamma angle, which, when reaching 90°, is critical for locking of the humeral head behind the glenoid.^11^ In our group, the gamma angle representing the posterior edge of RHSD appears to be more consistent thoroughout measured group and varied only from 88° to 98° (mean 92.4°). On the contrary, the beta angle, representing the anterior edge of RHSD, varied more widely, from 30° to 56° (mean 39.4°). The size of the defect represented with alpha angle varied significantly as well, from 36° to 64° (mean 53.0°). Concluding that the posterior border of the defect varied only minimally with different defect sizes (there was no correlation found between alpha and gamma angles), the defect size (alpha angle) increases mainly ventrally at the expense of the beta angle. We found an inverse correlation between the alpha and the beta angle, indicating that the enlargement of the defect is directed mainly anteriorly, toward the lesser tubercle. It may be either as a direct result of the injury or due to its progression over time.

The treatment method for locked dislocations depends on the size of the defect and on the time delay from the injury. A distinction is made between the anatomical reconstruction using bone grafts and the non-anatomical reconstruction using the transfer of the subscapularis muscle tendon or the lesser tuberosity into the defect. Posterior labral-capsular reconstruction in open procedures is rarely needed.^8^ Anatomical reconstruction involves repairing the defect while retaining its intra-articular position. It is mainly used for acute cases, which range from 25% to 40% of the humeral head joint area, where the reconstruction is possible by elevating of the impacted osteochondral fragment and supporting it with an autologous bone graft.^15^^,^^16^ Bock et al.^17^ performed the elevation in 6 patients within 14 days of injury and in 1 patient 180 days of dislocation. The extent of defects ranged from 30% to 45% of the joint area. Reoperation was required in only 1 patient, with the humeral head lesion of 40% of the surface, treated 180 days after the injury. Anatomical reconstructions of RHSD by elevation of the impressed bone supported with bioabsorbable screws can be performed using minimally invasive approach as well.^18^ Assom supported the elevated impressed fragment with 2 absorbable screws through the rotator interval in 2 patients. These patients with defects of 40% and 50% had good results after 26 months.^19^ In early diagnosed patients, the osteochondral fragment can also be acutely elevated retrograde using a trans-humoral tunnel. A retrograde procedure using balloon kyphoplasty with the bone cement in an acute case was performed by Ratner et al.^20^ This procedure has been reported, as an open surgery, in 3 patients.^21^ More suitable method to perform the anatomical reconstruction in neglected and chronic cases with the extensive bone loss, ranging from 25% to 50% is an open reduction and autogenous or allogeneic osteochondral bone grafting of the RHSD.^8^^,^^22^ The advantage of an autologous graft is its feasibility in emergency cases when the allograft is not available.^15^ An alternative to the iliac crest bone is the lateral clavicle graft.^23^ The most commonly used allogeneic grafts are taken from frozen or freshly autoclaved femoral heads.^24^ A talus bone graft is another option for the reconstruction.^25^^,^^26^ Gerber and Lambert reported good results using the femoral head allograft in 4 patients after 68 months of follow-up in defects with the sizes of 20% − 40%.^8^ Martinez et al.^27^ used frozen humeral allografts in 6 patients to fill defects above 40% with a delay from 7 to 8 weeks after the injury. In 2 cases, they observed graft collapse and the development of osteoarthritic changes after 62.2 months, and in 2 other cases after 122 months. Arthroplasty was needed in 3 of 6 patients, after 8 − 10 years. Diklic et al.^28^ observed 1 graft necrosis and 7 graft deformities among 13 patients after 54 months, with a mean CM score of 86.8 points. These observations suggest long-term complications in more than half of patients using allografts. More favourable results were observed in 10 patients, reconstructed with iliac crest autograft or allograft, and the mean follow-up period was 22.6 months, where Mi et al.^29^ reported healing in all patients with significant improvement of range of motion and CM score without any complications. An arthroscopic autologous iliac crest grafting was reported as well.^30^

The utilisation of allografts in our department is limited to irradiated femoral head allografts and morselized impaction bone grafting. The iliac bone autograft is linked to donor side pain and overall morbidity. Due to uncertain results with anatomical reconstructions in chronic defects, we have decided to use non-anatomical reconstructions for our patients. The basic principle of non-anatomical reconstructions is an externalisation of the RHSD outside the joint space. We prefer these procedures due to the advantage of using one's own tissue and the use of a single approach for the open reduction and the defect reconstruction.

In McLaughlin's original description, the subscapularis muscle tendon was separated from the lesser tuberosity and inserted into the defect using bone sutures.^31^^,^^32^ Hawkins et al.^33^ described a modification with an osteotomy of the lesser tubercle and its transfer into the lesion. These techniques are traditionally indicated for minor defects, usually up to 30% of the humeral head joint area.^8^ Some authors advocate their use in larger lesions covering up to 50% of the surface.^7^^,^^16^^,^^32^, ^33^, ^34^ Shams et al.^2^ describe 11 patients operated on for an average of 9 weeks after the injury by a transfer of the lesser tubercle with non-absorbable suture fixation.

Using this technique, good results were reported in patients with defects between 30% and 40%, resulting in a CM score of about 85 points, obtaining good abduction and external rotation.^12^^,^^35^ Compared to these results, we achieved an average CM score of 76 points in our patients with the lesser tubercle transfer. We did not observe shoulder redislocation in any patient. Except for 1 patient, the internal rotation of the shoulder joint resulted in a good range of motion, allowing the placement of the hand up to the lumbosacral area without signs of discomfort or instability. The modified McLaughlin procedure was performed by Hart et al.^36^ on 17 patients with neglected posterior dislocations, resulting in an active elevation of 113° on average. Kabelka et al.^37^ reported very good short-term results with the mean CM score of 86.5 points in 7 patients, after open McLaughlin or modified McLaughlin procedure. Demirel et al.^12^ obtained satisfactory results with a CM score of 85 points and no evidence of osteoarthritis in the follow-up from 12 to 67 months.

The outcome may be downgraded due to delay from the injury, as reported by Cohen et al.^38^ Poor function and glenohumeral joint degeneration were observed in 2 patients with delay from injury over 6 months in the case-series of 10 patients.

Less invasive procedures have become more popular for the treatment of RHSD in both acute and chronic indications. These are most often based on non-anatomical reconstructions inserting the subscapularis muscle tendon into the Hill-Sachs defect using an open approach or arthroscopically.^31^^,^^39^^,^^40^ Romano et al.^41^ reported 12 patients with a CM score of 94 points after an arthroscopic McLaughlin procedure. This anchoring and tenodesis of the subscapularis muscle is indicated in defects under 40% of the articular surface area of the humeral head.^42^, ^43^, ^44^ Alternatively, it is possible to fill the lesion with the medial glenohumeral ligament.^45^ The advantage of the arthroscopy is the possibility of reconstruction of the posterior labral Bankart's lesion at the same time, if necessary.^46^, ^47^, ^48^ Despite promising results with arthroscopic treatment of RHSD, we did not indicate this method for our patients. Due to concerns about the possible failure of a remplissage with soft tissue and the complexity of the arthroscopic technique, we opted for an open procedure with a good functional outcome. To advocate our decision, Ippolito et al.^49^ found no difference in 6 open versus 4 arthroscopic McLaughlin procedures, achieving a CM score over 80 points in both groups.

Rotational osteotomy of the humeral head is the reserve option instead of an endoprosthetic solution, when the defect reconstruction is not eligible, especially in young patients.^1^ Arthroplasty would be considered in defects exceeding 40% − 50% of the joint area, taking into account the age and complaints of the patient.^8^ Gavriilidis evaluated a group of 12 patients with large head defects over 45%, treated with hemiarthroplasty in 10 cases and in 2 cases with total shoulder replacement. The average postoperative CM score after 3 years was 59.4 points.^50^ Popelka reports excellent results in 2 patients with chronic reverse Hill-Sachs lesions over 45% of the cartilage area and treatment with total shoulder replacement at 1-year and 3-year follow-up.^51^ Despite good results with endoprostheses, in cases where degenerative changes are absent, this solution still presents a significant intervention in the integrity of the shoulder joint, with a non-negligible risk of mechanical and infectious complications.

Our results in the group reconstructed with the lesser tubercle transfer ranged from excellent to good, with the CM score ranging 67 − 84 points. The greatest improvement was achieved by obtaining the external rotation. We did not notice any infectious or neurovascular complications in the patients. Indications were found that the defect size is inversely proportional to the beta angle, and the defects increase in size towards the anterior margin.

The limitation of the study is a small cohort of patients. Otherwise, these injuries are rare at all, and the occurrence of overlooked and neglected cases is decreasing, thanks to the use of CT and MRI.

In conclusion, based on our own observations, using an autologous tissue for the reconstruction of the RHSD of the humeral head appears to be a safe and reasonable solution. We assume that even in larger inverted defects of around 30% − 40% of the humeral head cartilage, the transfer of a lesser tubercle is a suitable solution with a high chance of excellent functional results.

## CRediT authorship contribution statement

**Ilja Chandoga:** Conceptualization, Data curation, Writing – original draft, Writing – review & editing. **Dávid Debnár:** Writing – original draft, Writing – review & editing. **Katarína Chandogová:** Writing – original draft, Writing – review & editing. **Miroslav Kilian:** Conceptualization, Data curation, Writing – original draft, Writing – review & editing.

## Ethical statement

The authors obtained written informed consent from all the patients. All procedures carried out in studies involving human participants complied with the ethical principles established by the institutional and/or national research committee, as well as with the Declaration of Helsinki and its subsequent revisions or equivalent ethical standards.

## Funding

The authors received no specific grant from any funding agency in the public, commercial, or not-for-profit sectors.

## Declaration of competing interest

The authors state that they had no interests which might be perceived as posing a conflict or bias.

## Data Availability

The data that support the findings of this study are available on request from the corresponding author. The data are not publicly available due to privacy or ethical restrictions.
